# Computational analysis of crosstalk between transcriptional regulators and RNA-binding proteins suggests mutual regulation of polycomb proteins and SRSF1 influencing adult hippocampal neurogenesis

**DOI:** 10.1007/s44192-023-00034-5

**Published:** 2023-03-06

**Authors:** M. J. Nishanth, Shanker Jha

**Affiliations:** 1 Department of Biotechnology, School of Lifesciences, St Joseph’s University, Bengaluru, India; 2 School of Chemical and Biotechnology, SASTRA Deemed University, Thanjavur, India

**Keywords:** Adult hippocampal neurogenesis (AHN), RNA-binding proteins (RBPs), SRSF, Polycomb complex, Post-transcriptional regulation, Physical exercise

## Abstract

**Background:**

Adult hippocampal neurogenesis (AHN) is a clinically significant neural phenomenon. Understanding its molecular regulation would be important. In this regard, most studies have focused on transcriptional regulators (TRs), epigenetic modifiers, or non-coding RNAs. RNA-binding proteins (RBPs) have emerged as dominant molecular regulators. It would be significant to understand the potential cross-talk between RBPs and TRs, which could influence AHN.

**Methods:**

The present study employed computational analyses to identify RBPs and TRs regulating AHN, followed by the analysis of their interaction networks and detection of hub proteins. Next, the potential mutual regulation of hub TRs and RBPs was analyzed. Additionally, hippocampal genes differentially expressed upon exercise were analyzed for potential regulation by the identified TRs and RBPs.

**Results:**

105 TRs and 26 RBPs were found to influence AHN, which could also form interactive networks. Polycomb complex proteins were among the TR network hubs, while HNRNP and SRSF family members were among the hub RBPs. Further, the polycomb complex proteins and SRSF1 could have a mutual regulatory relationship, suggesting a cross-talk between epigenetic/transcriptional and post-transcriptional regulatory pathways. A number of exercise-induced hippocampal genes were also found to be potential targets of the identified TRs and RBPs.

**Conclusion:**

SRSF1 may influence post-transcriptional stability, localization, and alternative splicing patterns of polycomb complex transcripts, and the polycomb proteins may in turn epigenetically influence the SRSF1. Further experimental validation of these regulatory loops/networks could provide novel insights into the molecular regulation of AHN, and unravel new targets for disease-treatment.

**Supplementary Information:**

The online version contains supplementary material available at 10.1007/s44192-023-00034-5.

## Introduction

Adult hippocampal neurogenesis (AHN; the process of generation of new neurons in the hippocampus and their integration into neural circuitry in adulthood) is implicated in key cognitive processes including memory encoding, affective behaviours, and mood regulation [1, 2]. Importantly, recent studies have shown psychiatric illnesses to be associated with impaired adult neurogenesis and hippocampal plasticity [3], highlighting the importance of AHN in normal brain functioning. Enhanced neurogenesis could rescue memory impairments in Alzheimer’s disease [4], while compromised AHN due to aging and neurodegenerative diseases was reported to potentially lead to hippocampal dysfunction [3]. In addition, exercise is a known positive regulator of AHN; voluntary wheel-running enhances AHN in experimental rodent models [1, 5, 6]. Exercise is known to upregulate the circulating levels of neurotrophic factors such as brain-derived neurotrophic factor (BDNF) and vascular endothelial growth factor (VEGF), which in turn enhance gliogenesis, neurogenesis, synaptogenesis, and angiogenesis. As a result, grey matter and white matter volumes, neural activity, receptor activity, and cerebral blood flow are increased, leading to improved cognitive function [7]. Exercise could inhibit the age-related reduction of neurogenesis, which was associated with better cognitive performance compared to sedentary individuals [8]. Sedentary lifestyle along with other factors may be associated with dentate gyrus astrocyte dysfunction as well as impaired learning and memory [9]. Thus, a holistic understanding of the molecular regulation of AHN would be of scientific and therapeutic importance. In this regard, unravelling the regulatory factors influencing AHN could provide potential novel cues towards manipulation of AHN.

With respect to molecular regulation of AHN, existing research has largely focused on transcriptional regulators (TRs), epigenetic factors, and non-coding RNAs [10–12]. RNA-binding proteins (RBPs) are a dominant class of molecular regulators known to influence multiple processes (such as microRNA-mediated control [13], alternative splicing [14], and editing [15]). RBP-mediated gene expression regulation is reported to be especially important in brain, and several RBPs are known to be critical modulators of neural stem cell growth and functioning. Embryonic lethal abnormal vision (ELAV), Pumilio (Pum), Musashi (Msi), Fragile X-related proteins (FMRP) are some of the known principal regulators of embryonic neural stem cells [16]. However, our understanding of the RBP-mediated regulation of AHN remains poorly understood. RBPs can also control transcriptional regulators, forming regulatory loops which could have diverse effects on the downstream genes. Owing to the dominant molecular control exerted by RBPs, it would be imperative to understand their effects on regulation of AHN. To this end, the present study employed computational approaches to identify RBPs forming regulatory hubs potentially influencing AHN-related genes, and also their transcriptional regulators.

In the present study, genes known to influence AHN (obtained through Mammalian Adult Neurogenesis Gene Ontology; MANGO database [17]) were analyzed to identify functional regulators influencing their transcription via cis-regulatory elements, and also the RBPs having overrepresented binding sites within their UTRs. Since TRs and RBPs are known to function as multi-protein complexes, interaction networks were identified within the TRs and RBPs potentially influencing AHN. Subsequently, hub proteins having significantly high interconnections within the protein networks were detected. Further, potential mutual-regulation of the hub TRs and RBPs was studied. As a result, SRSF1 was found to be a potential regulator of AHN. In addition, SRSF1 could also modulate the polycomb complex proteins, which could in turn influence AHN via epigenetic mechanisms. Further, polycomb proteins may also influence the expression of *SRSF1*. Thus, SRSF1 and polycomb complex proteins may form regulatory loops, influencing AHN. Next, we adopted a meta-analysis approach to study the possible effects of these regulators on hippocampal gene expression changes in response to exercise. Interestingly, exercise-induced differential gene expression patterns were found to be potentially influenced by the identified hub TRs and RBPs. To the best of our knowledge, the present report is the first study to examine the molecular cross-talk between multiple regulatory factors which could influence AHN. Thus, the present findings could form a foundation for future experiments to understand the effects of multiple regulatory factors and their cross-talk, on AHN. Further studies in this regard could provide novel molecular cues to modulate AHN towards cognitive well-being.

## Methods

### Identification of TRs and RBPs potentially influencing AHN-related genes

The genes associated with AHN were retrieved from The Mammalian Adult Neurogenesis Gene Ontology (MANGO) database (mango.adult-neurogenesis.de) (v 3.2) [17]. This database consists of 397 genes implicated in AHN. These genes were analysed to identify overrepresented binding sites for transcriptional regulators (TRs) and RNA-binding proteins (RBPs) within the genomic 5′ upstream regions and the untranslated regions (5′ and 3′UTRs) respectively. Overrepresented TR binding sites (TRBSs) (Irwin-Hall p-value < 0.01) were identified using BARTweb tool (bartweb.org/) (v 2.0) [18] against mouse (mm10) database. BARTweb identifies transcriptional regulators associated with the genes of interest. The TRs having Irwin-Hall p-value < 0.01 could have a significant impact on the regulation of considered genes [18]. RBPs having enriched binding sites in the UTRs of the AHN-related genes were identified using the Regulatory elements enrichment tool of AURA (aura.science.unitn.it/) (v 2.5.2) [19], against *Mus musculus* (mm10) database. In addition, the AHN-related genes from MANGO database were compared with known TRs and RBPs of mouse, to identify TRs and RBPs known to be involved in AHN. Gene names of known TRs were obtained from AnimalTFdb (v 3.0) (http://bioinfo.life.hust.edu.cn/AnimalTFDB/#!/download) [20], while the RBPs were retrieved from three databases, RBPDB (v 1.3.1) (rbpdb.ccbr.utoronto.ca/) [21], RBPmap (v 1.2) (rbpmap.technion.ac.il/) [22], and Transite (https://transite.mit.edu/) [23].

### Analysis of protein–protein interactions and identification of hub proteins

The potential interactions among the identified TRs and RBPs were analysed via STRING, using default settings (minimum interaction score = 0.4; this moderate cut-off score increases the coverage of the possible protein interactions, and prevents the overestimation of their precision [24]). Interactions were retrieved through textmining, experiments, databases, co‑expression, neighbourhood, gene fusion, and co‑occurrence. The interaction network obtained from STRING was exported to Cytoscape [25] (v 3.8.0) and the top 10 hub genes were identified via cytohubba [26] plug-in, through the eleven analysis methods of the tool (maximal clique centrality/MCC, density of maximum neighbourhood component/DMNC, maximum neighbourhood component/MNC, degree, closeness, betweenness, radiality, stress, EcCentricity, BottleNeck, and Edge Percolated Component). However, the hub nodes identified via MCC were considered for further analysis, since this method was reported to be more precise in predicting essential hub nodes [26].

### Mutual-regulation of TRs and RBPs potentially influencing AHN

The hub proteins within the TR and RBP interaction networks were independently analyzed, to identify their potential mutual-regulation. For this purpose, the hub TR genes were analyzed to identify their RBP regulators using the Regulatory elements enrichment tool of AURA (aura.science.unitn.it/) (v2.5.2) [19]. Also, TRs regulating the hub RBP genes were identified using BARTweb tool (bartweb.org/) (v2.0) [18] against mouse (mm10) database.

The predicted binding sites for the RBP, SRSF1 on the 5′ and 3′UTRs of selected TRs were identified via RBPmap (v1.2) (rbpmap.technion.ac.il/) [22]. The UTR sequences were retrieved from NCBI nucleotide database, by comparing the coding sequence (CDS) and mRNA sequences of the considered TR genes. The nucleotide sequence upstream to ATG/start codon and downstream to stop codon were retrieved as 5′ and 3′UTRs, respectively.

Further, in order to understand the implications of the identified TRs and RBPs on AHN, we examined the genes on MANGO database to study their potential regulation by the identified proteins. Thereby, potential targets of these regulators were identified among the AHN-related genes curated onto MANGO. The enrichment of histone marks on AHN-related genes was analyzed using AnnoMiner (http://chimborazo.ibdm.univ-mrs.fr/AnnoMiner/annominer.html), against mouse (mm10) database.

### Analysis of the influence of the identified hub TRs and RBPs on exercise-induced differential gene expression in adult mouse hippocampus

Exercise is a robust enhancer of AHN. We employed a meta-analysis approach to study the potential involvement of the identified regulatory proteins on exercise-mediated gene expression changes in hippocampi of adult mice. For this purpose, exercise-induced differential gene expression data from our previous study [27] was utilized. Analysis of differential gene expression patterns using publicly available RNAseq datasets has been described in detail previously [27]. Briefly, differentially expressed genes (DEGs) in response to physical exercise in mouse hippocampal samples were identified using OneStopRNAseq webserver (https://mccb.umassmed.edu/OneStopRNAseq/index.php). The tool identifies DEGs employing DESeq2 package. This analysis was conducted using the default cut-off levels (adjusted p value of 0.05, and logarithmic fold change of 0.585). DESeq2 adjusts the Wald test p values for multiple testing using the Benjamini and Hochberg procedure [28, 29]. In the present study, the exercise-induced DEGs were compared with known target genes of the RBPs and TRs of interest. Thus, the present study indexed the genes which could be regulated by the identified TRs and RBPs in response to exercise, within the hippocampal tissues of adult mice. Exercise-induced differentially-expressed genes were analyzed to identify potential targets of SRSF1 (RBP) and H3K27me3 methylation (TRs). AHN-related genes (from MANGO) were found to have 83 and 67 genes which are targets of SRSF1 and H3K27me3, respectively. These 67 and 83 genes were compared with individual lists of exercise-induced DEGs, yielding the exercise-influenced AHN genes which could be targets of SRSF1 and H3K27me3 activity.

**Fig. 1:**
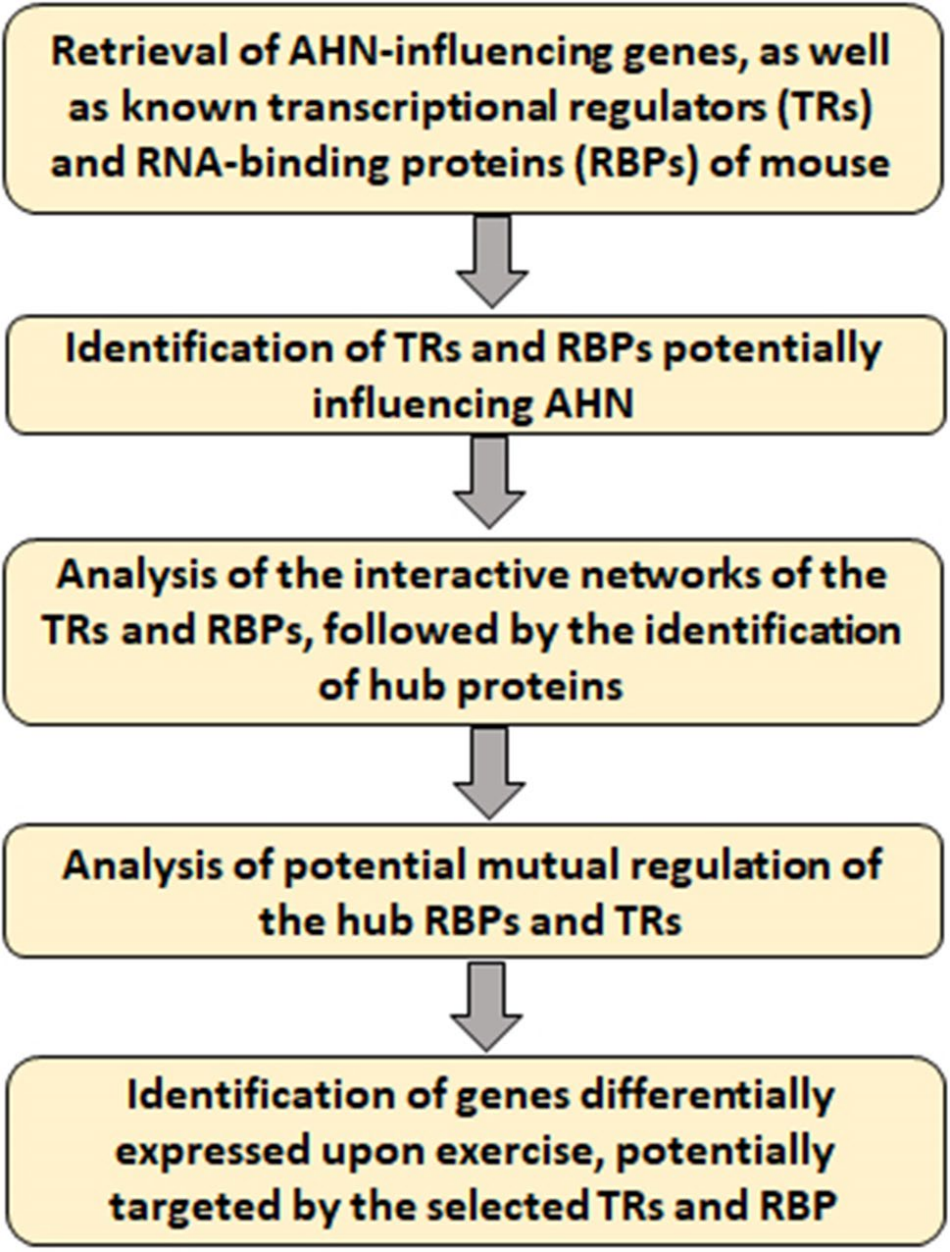
Work-flow of the present study. AHN-related genes were retrieved from Mammalian Adult Neurogenesis Gene Ontology (MANGO) database, followed by identification of transcriptional regulators and RNA-binding proteins influencing these genes. Subsequently, inter-molecular interactions of these proteins were analyzed and the hub-proteins were identified. Further, potential mutual-regulation of the hub proteins was studied, which showed the interaction between transcriptional and post-transcriptional regulators influencing AHN

The work-flow of the present study has been given in Fig. 1.

## Results

### Identification of TRs and RBPs potentially influencing AHN-related genes

**Fig. 2:**
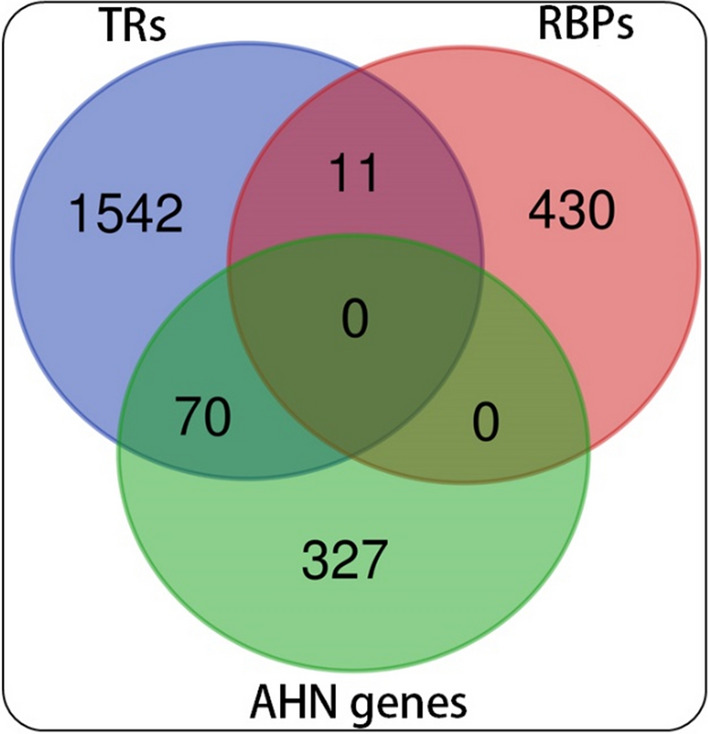
Number of transcriptional regulators (TRs) and RNA-binding proteins (RBPs) associated with adult hippocampal neurogenesis (AHN). Genes associated with AHN (obtained from MANGO database) were compared with known TRs and RBPs retrieved from respective databases

The AHN-related genes curated onto MANGO database were compared with known TRs and RBPs of mouse, to identify TRs and RBPs known to be involved in AHN, identifying 70 TRs. No RBPs were found to be associated with the database, indicating a lack of studies on RBP-mediated regulation of AHN (Fig. 2). The AHN-related TRs (identified from MANGO database), were combined with those TRs obtained via BARTweb (TRs having overrepresented binding sites on the 5′upstream regions of AHN-related genes), which yielded 105 TRs potentially involved in the regulation of AHN. The AHN-related genes, TRs, and RBPs considered in the present study are given in Supplementary File 1.

The analysis of RBPs having enriched binding sites in the UTRs of the AHN-related genes identified 26 RBPs which could influence AHN (Elavl1, Tardbp, Nova2, Srsf1, Ezh2, Srsf3, Srsf2, Dnd1, Elavl2, Ptbp2, Rbm8a, Apobec1, TIS, Elavl4, m6A, Ago, Srsf4, Ago2, Rbm3, Dgcr8, Ddx58, Fxr1, Celf1, Mbnl2, Fmr1, Cpeb1).

### Interaction networks of TRs and RBPs

The potential interactions among the identified TRs and RBPs were analysed through STRING, using default settings, which revealed an extensive network with 104 nodes and 834 edges. The protein interactors of the 26 RBPs were studied using string (v 11.5) (https://string-db.org/) and MIPPIE (v 1.0) (cbdm-01.zdv.uni-mainz.de/ ~ galanisl/mippie/index.php) databases. For this purpose, all the experimentally-validated protein interactors of the 26 RBPs were identified, followed by screening and selection of RBPs. Totally, 123 RBPs were identified, forming an interactive network having 121 nodes and 2110 edges (Supplementary Fig. 1–2).

### Identification of hub-proteins

**Fig. 3:**
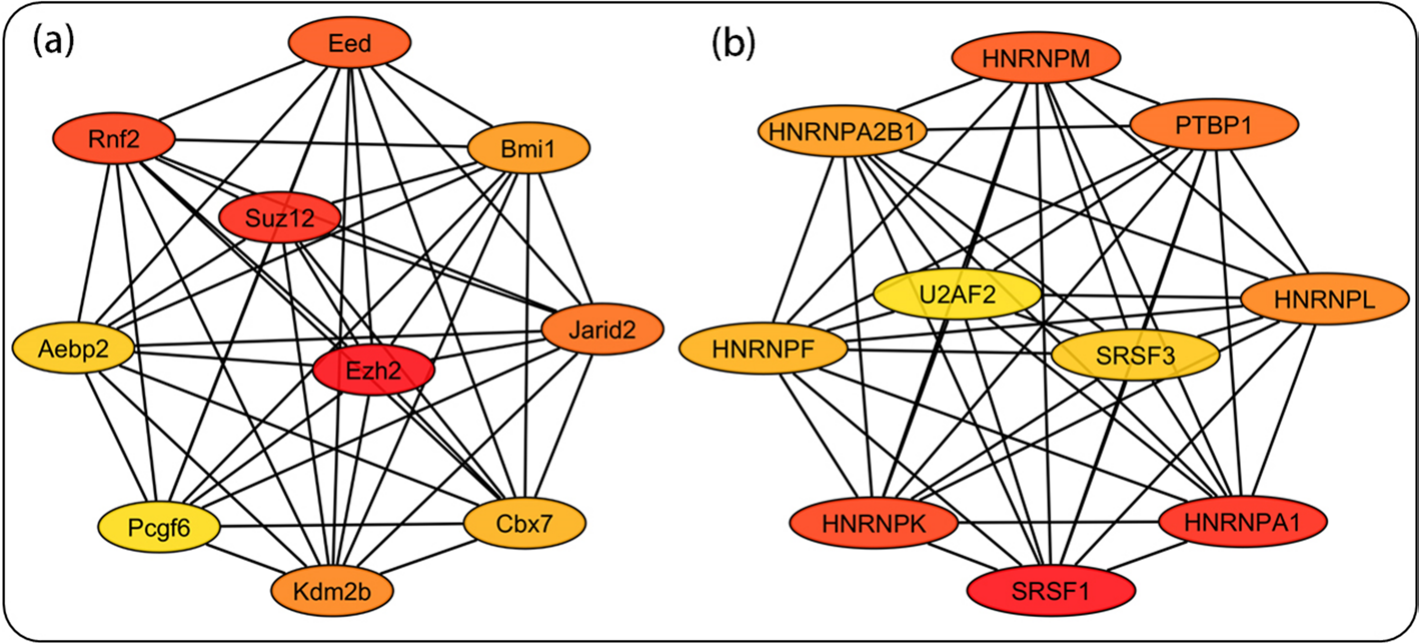
Hub proteins of transcriptional regulator (TRs) and RNA-binding protein (RBPs) networks. The top ten hub proteins of the TR (**a**) and RBP (**b**) networks having extensive interconnections are shown

The top 10 hubs having significantly higher inter-connections with other proteins of the network were identified. MCC-based analysis through CytoHubba identified proteins belonging to the polycomb complex involved in epigenetic modifications to be among the hub TRs, while heteronuclear ribonucleoprotein (HNRNP) and serine/arginine-rich splicing factor (SRSF) family members formed the RBP hubs. These results are shown in Fig. 3 and Table 1. The top ten hub nodes identified through other methods of CytoHubba plug-in are given in Supplementary File 2.

**Table 1:** Hub TRs and RBPs potentially influencing AHN

Rank	TR	RBP
1	Ezh2	Srsf1
2	Suz12	Hnrnpa1
3	Rnf2	Hnrnpk
4	Eed	Hnrnpm
5	Jarid2	Ptbp1
6	Kdm2b	Hnrnpl
7	Bmi1	Hnrnpa2b1
8	Cbx7	Hnrnpf
9	Aebp2	Srsf3
10	Pcgf6	U2af2

### Analysis of mutual-regulation of the hub TRs and RBPs, and their influence on potential regulation of AHN-related genes

The UTRs of the hub TR genes were found to possess overrepresented binding sites for SRSF1, SRSF2, and TARDBP. Notably, SRSF1 was also detected among the 10 hub RBPs involved in the regulation of AHN genes. The analysis of TRs potentially regulating the hub RBP genes identified 42 TRs with enriched binding sites in the genomic 5′upstream regions of the hub RBPs. Among these, CBX7, EZH2, KDM2B, RNF2, and SUZ12 (polycomb repressive complex proteins) were also identified, which were found to be hub TRs involved in regulating AHN-related genes. These observations point toward potential molecular regulatory loops formed by epigenetic/transcriptional and post-transcriptional processes, which could affect the target transcripts and the associated biological processes.

Since SRSF1 was identified to be involved in the regulation of (a) AHN-related genes, and also (b) TRs influencing the AHN genes, we further analyzed the number of SRSF1 binding sites on the UTRs of the five TRs which were a part of TRs hub and also found to regulate the hub RBPs. The number of SRSF1 binding sites predicted in the UTRs of these five transcripts are given in Table 2.

**Table 2:** Number of SRSF1 binding sites on the UTRs of hub TRs (polycomb complex members) potentially regulating the hub RBPs

S. No	Transcript	3′UTR	5′UTR
1	*Cbx7*	8	None
2	*Suz12*	None	None
3	*Ezh2*	None	None
4	*Kdm2b*	4	None
5	*RNF2*	1	None

The AHN-related genes on MANGO database were studied to analyze their potential regulation by SRSF1 and the selected polycomb proteins. As a result, SRSF1 was found to potentially regulate 83 of the 397 AHN genes (20.91%). Since the identified polycomb proteins are known modulators of histone modifications, we analyzed the enrichment of histone marks on AHN-related genes. It was found that H3K27me3 marks were enriched in these genes, suggesting the involvement of polycomb complex proteins in their regulation. 67 genes among the 397 MANGO genes were found to have an enriched propensity for H3K27me3 modifications. Notably, BDNF, VEGFA, VGF, IGF2, and NGF were found to be targets of SRSF1 activity and/or H3K27me3 modifications. The complete list of potential target genes of SRSF1 and polycomb proteins, which could influence AHN is provided in Supplementary File 3.

### Exercise-induced hippocampal gene expression changes: expression and effects of the identified TRs and RBPs

The expression pattern of the six identified regulators (SRSF1, CBX7, SUZ12, EZH2, Kdm2b, and Rnf2) in response to physical exercise in rodent models were analyzed using the differential gene expression data from our previous meta-analysis [27]. SRSF1, SUZ12, and Rnf2 were found to be downregulated in the dataset GSE39697 (outer granule cell layer of the hippocampus), while the other datasets showed no difference in their expression levels. Thus, physical exercise may not cause a widespread change in the expression levels of these regulatory factors. However, a number of other genes differentially expressed in response to exercise were found to be potential targets of SRSF1 and H3K27me3 (Table 3).

**Table 3:** Exercise-induced differentially expressed genes potentially regulated by SRSF1 and H3K27me3

S.No	Dataset and experiment	Total no. of DEGs	Genes differentially expressed upon exercise	
			SRSF1 targets	H3K27me3 targets
1	GSE39697 4 d run vs. sedentary; GC layer	1177	Gadd45b, Cdkn1a, Ndel1, Pten, Htt, Ube3a, Casp3, Sparc, Dnm1l, Pcmt1, Pafah1b1, Ctnnb1	Fst
2	GSE39697 30 d run vs. sedentary; GC layer	2	None	None
3	GSE39697 4 d run vs. sedentary; SGZ	552	Gadd45b, Cdkn1a, Pafah1b1	None
4	GSE39697 30 d run vs. sedentary; SGZ	8	None	None
5	GSE86235; Runners vs. sedentary	249	Cdkn1a	Eomes, Vipr2, Slc12a5, Slc18a2
6	GSE142678; 27 d Run vs. sedentary; immature neurons	34	None	Grm2
7	GSE142678; 27 d Run vs. sedentary; dentate gyrus	803	Ccnd1	Akt1, Atf2, Btg1, Cdkn1b, Dbi, Per2, Rac1, Rhoa
8	GSE179081, Run vs. sedentary; dentate gyrus	13	None	None
9	GSE132930, Run vs. sedentary; hippocampus	818	Notch2, Per2	Eomes, Htr2c, Lepr, Trp73

## Discussion

AHN is implicated in mental illnesses and well-being through studies in animal models as well as humans [1–3]. A holistic understanding of the molecular regulation of AHN would provide potential cues to modulate the process and possibly offer new treatment options for mental illnesses. In this regard, most research so far has focused on the involvement of transcription factors, epigenetic modulators, micro-RNAs, and non-coding RNAs [10–12, 30]. RNA-binding proteins have emerged as a principal class of molecular regulators which could affect other regulatory factors [13, 14]. It would be important to understand the potential cross-talk between regulatory molecules across multiple steps of gene expression. To this end, the present work focused on understanding the potential interactions and mutual-regulation of transcriptional regulators and RBPs, to get insights into the molecular cross-talk between transcriptional and post-transcriptional regulatory pathways. A detailed analysis of genes implicated in AHN identified 105 TRs and 123 RBPs potentially involved in their regulation. These regulatory factors were found to form interactive networks, and the top 10 hub TRs and RBPs with extensive interactions were identified. Further analysis of these hub proteins revealed SRSF1 to be a potential regulator of the transcripts of polycomb proteins (*Cbx7*, *Suz12*, *Ezh2*, *Kdm2b*, *RNF2/RING1B*) through their UTRs. SRSF1 could potentially modulate alternative splicing as well as localization of the polycomb complex-encoding transcripts, which could further influence proliferation, differentiation, and survival of adult-born neurons by modulating histone methylation patterns and transcription of AHN-related genes. Thus, SRSF1 could play a dominant role in the maintenance of AHN. Further, transcription of *SRSF1* was shown to be possibly influenced by the polycomb complex. Thus, a potential regulatory loop influencing AHN across transcriptional and post-transcriptional levels was identified. In addition, AHN-related genes differentially expressed in response to exercise were also found to be potentially regulated by SRSF1 and the polycomb complex, suggesting their involvement in exercise-induced AHN. Notably, functionally important genes impacting the numbers, proliferation, and survival of adultborn neurons were identified to be potentially targeted by the identified TRs and RBPs. BDNF (an established regulator of AHN [31, 32]) was found to be a target of both SRSF1 and polycomb proteins. Fmr1, another known regulator of AHN [33] was also found to be a potential target of SRSF1. Thus, it would be interesting to analyze the molecular effects of these regulators on AHN, and their mechanisms.

Polycomb repressive complexes (PRCs) are a major class of epigenetic modulators functioning as multi-protein ensembles. They consist of two protein complexes, PRC1 and PRC2. PRC1 is involved in monoubiquitilation of histone H2A at Lys119 (H2AK119ub1), while PRC2 is known to mono-, di-, and tri-methylate Histone H3 (H3K27me1, H3K27me2, and H3K27me3) [34]. Among the five genes of PRC components considered in the present study, RING1B forms the catalytic core of PRC1, while CBX7 and KDM2B act as auxiliary proteins. SUZ12 and EZH2 are among the catalytic core components of PRC2 [34]. Cbx7, Suz12, and Ezh2 are involved in H3K9me3/H3K27me3 modifications, and Rnf2 is involved in H2AK119Ub modification, while Kdm2b is involved in demethylation of Lys in histone 3 [34]. Interestingly, a number of polycomb proteins are known to play critical roles in neural development [35]. The expression of EZH2 was reported in astrocytes which serve as neural stem cells in adult mouse subventrcular zone (SVZ), and the repression of Olig2, an EZH2 target, was reported to play an important role in AHN [36]. Expression of EZH2 in cultured murine astrocytes was reported to be associated with dedifferentiation of astrocytes and subsequent development of neurons and neurospheres [37]. EZH2 was also shown to be important for neuronal proliferation as well as stemness [38]. KDM2B is also a known critical regulator of neural stem cells. Loss of KDM2B was reported to be associated with impaired PRC1 recruitment resulting in derepression of genes involved in cell cycle regulation and apoptosis, leading to memory deficits [39]. CBX7 was reported to influence axon growth in embryonic murine cortical neurons and adult dorsal root ganglion neurons. Two important transcription factors, GATA4 and SOX11 are known targets of CBX7, and the knock-down of CBX7 was associated with enhanced axon growth ability of neurons [40]. These reports support the present findings, wherein the PRC complex proteins were found to form hubs of transcriptional regulatory interactions influencing AHN-related genes.

RBPs regulate multiple aspects of post-transcriptional processes (including alternative splicing, mRNA export, and transcript stability regulation) across neuronal developmental stages during embryonic and adult neurogenesis [27, 41]. CPEB3, HuD, HuR, FXR2, Msi are among the RBPs known to potentially influence cell proliferation, differentiation, migration, and maturation of adult-born neurons [41]. The SRSFs are a major class of RBPs, which interact with other RBPs such as HNRNPs, and regulate alternative splicing patterns leading to specific cellular and tissue developmental patterns [42]. SRSF1 is a well-studied SR family protein involved in several processes including alternative splicing, mRNA export, translation, and decay. SRSF1 is also known to play important roles in cellular fate determination and tissue development. SRSF1 was reported to be essential for regulation of gene networks involved in thymocyte differentiation, proliferation, and apoptosis [43]. Interestingly, SRSF1 was also reported to be associated with neurodegeneration in *Drosophila*, suggesting its potential importance in the maintenance of neuronal homeostasis [44]. These reports underscore the significance of SRSF1 in development, maintenance, and functioning of several cell-types including neurons. Further research to decipher the functional implications of SRSF1 on AHN via polycomb complex and also other pathways could provide valuable insights into the molecular regulation of AHN. However, the following conceptual limitations should be considered: the present study is based on the information curated onto the considered databases. Also, the hub nodes within the regulatory protein interactive networks were identified using MCC algorithm, reported to be more accurate than the other algorithms. Considering other algorithms might reveal additional hub nodes and regulatory interrelations.

## Conclusions

**Fig. 4:**
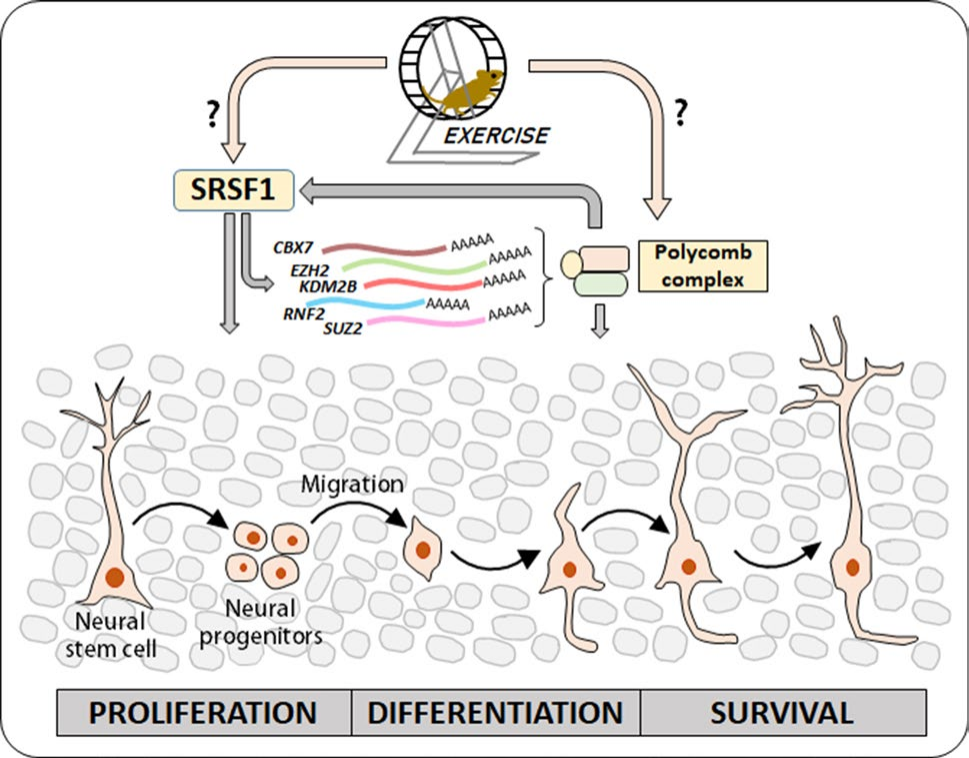
A model of mutual-regulation of SRSF1 and polycomb gene transcripts influencing AHN. SRSF1 could be involved in post-transcriptional regulation of polycomb gene transcripts, and the polycomb complex could in turn regulate the transcription of SRSF1. These proteins could also influence multiple aspects of AHN. Further, SRSF1 and polycomb complex proteins could influence exercise-induced AHN by altering their target gene expression patterns. Hence, potential cross-talk between transcriptional and post-transcriptional regulators may influence AHN

AHN is a clinically important neural process. A holistic understanding of the molecular regulation of AHN would be necessary towards the therapeutic interventions employing/targeting AHN. In this regard, the existing research reports have largely focused on individual regulatory factors such as transcriptional and epigenetic modulators, and non-coding RNAs. RBPs are now known to play predominant roles in gene expression regulation. The influence of RBPs, and also the potential interplay between multiple regulatory factors which could influence AHN are yet to be understood. To this end, the present work identified the TRs and RBPs potentially influencing AHN. The molecular interaction networks formed by these regulatory proteins were studied. Subsequently, the possible mutual-regulation of the hub RBPs and TRs was examined. As a result, SRSF1 was found to be potentially regulating multiple AHN-related genes, and also epigenetic modulators known to influence the transcription of AHN-related genes (Cbx7, Suz12, Ezh2, Kdm2b, and RNF2). In addition, the analysis of exercise-induced gene expression changes (known to upregulate AHN) suggested an influence of the identified regulatory proteins, on AHN (Fig. 4). Thus, future experimental studies to unravel the regulation of AHN through SRSF1 could provide novel insights into the molecular mechanisms underlying AHN, and possibly reveal novel therapeutic targets to modulate AHN. In this regard, RNA–protein and protein–protein interaction studies, as well as biochemical studies to understand the potential involvement of phase separation and ribonucleoprotein complexes could provide experimental insights into the regulatory mechanisms of AHN. A complete understanding of the regulatory factors controlling AHN could suggest candidate molecules which could be targeted to influence AHN and neurological conditions associated with this process.

## Supplementary Information

Below is the link to the electronic supplementary material.

Supplementary file1 (XLSX 58 KB)

Supplementary file2 (XLSX 12 KB)

Supplementary file3 (XLSX 20 KB)

Supplementary file4 (DOCX 6480 KB)

## Data Availability

All the relevant data pertaining to the manuscript are available within the manuscript and the supplementary material.
