# Supplementary material for: Computational analysis of crosstalk between transcriptional regulators and RNA-binding proteins suggests mutual regulation of polycomb proteins and SRSF1 influencing adult hippocampal neurogenesis
Source: Discov Ment Health. 2023 Mar 6;3(1):7. doi: 10.1007/s44192-023-00034-5 (PMC10501017; doi:10.1007/s44192-023-00034-5)
Supplement: Supplementary file 4 — Supplementary file4 (DOCX 6480 KB) [file 44192_2023_34_MOESM4_ESM.docx]

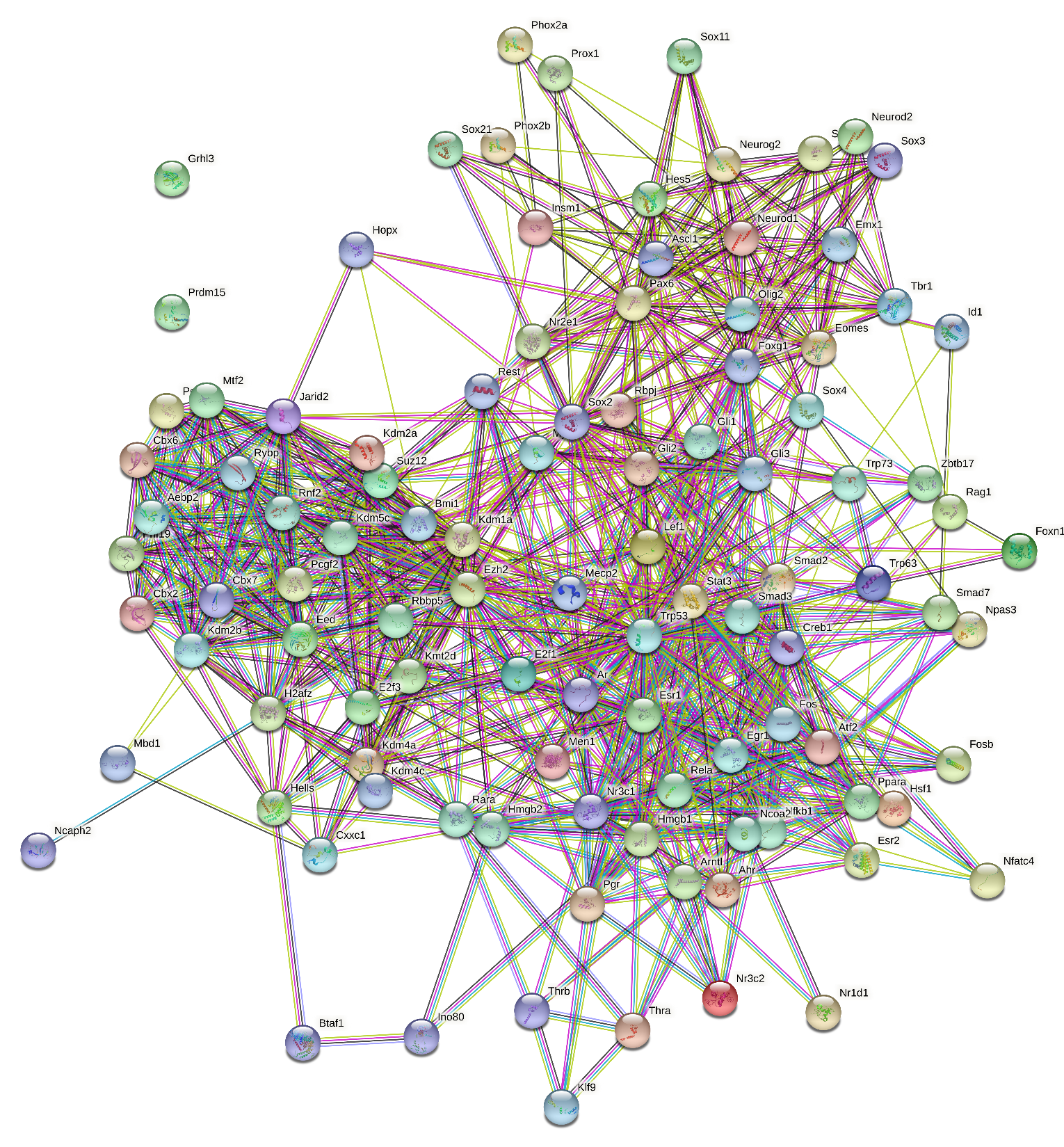


**Supplementary Fig. 1 Interactive networks of the identified TRs** The network consists of 104 nodes and 834 edges, obtained from STRING, using default settings.


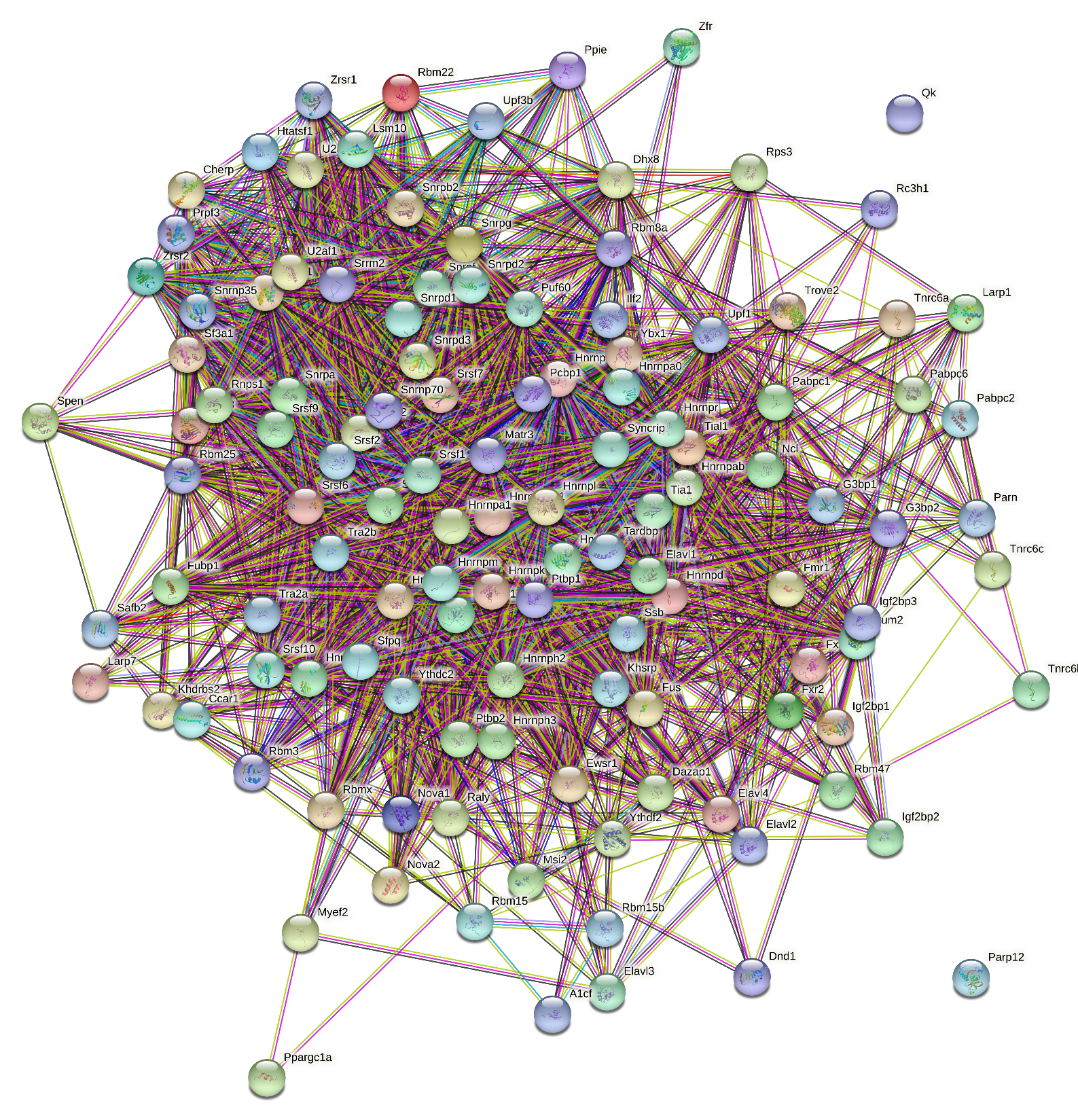


**Supplementary Fig. 2 Interactive networks of the identified RBPs** The network consists of 121 nodes and 2110 edges, obtained from STRING, using default settings.
